# Navigating the Interface Between Landscape Genetics and Landscape Genomics

**DOI:** 10.3389/fgene.2018.00068

**Published:** 2018-03-13

**Authors:** Andrew Storfer, Austin Patton, Alexandra K. Fraik

**Affiliations:** School of Biological Sciences, Washington State University, Pullman, WA, United States

**Keywords:** landscape genomics, landscape genetics, local adaptation, selection, spatial analyses

## Abstract

As next-generation sequencing data become increasingly available for non-model organisms, a shift has occurred in the focus of studies of the geographic distribution of genetic variation. Whereas landscape genetics studies primarily focus on testing the effects of landscape variables on gene flow and genetic population structure, landscape genomics studies focus on detecting candidate genes under selection that indicate possible local adaptation. Navigating the transition between landscape genomics and landscape genetics can be challenging. The number of molecular markers analyzed has shifted from what used to be a few dozen loci to thousands of loci and even full genomes. Although genome scale data can be separated into sets of neutral loci for analyses of gene flow and population structure and putative loci under selection for inference of local adaptation, there are inherent differences in the questions that are addressed in the two study frameworks. We discuss these differences and their implications for study design, marker choice and downstream analysis methods. Similar to the rapid proliferation of analysis methods in the early development of landscape genetics, new analytical methods for detection of selection in landscape genomics studies are burgeoning. We focus on genome scan methods for detection of selection, and in particular, outlier differentiation methods and genetic-environment association tests because they are the most widely used. Use of genome scan methods requires an understanding of the potential mismatches between the biology of a species and assumptions inherent in analytical methods used, which can lead to high false positive rates of detected loci under selection. Key to choosing appropriate genome scan methods is an understanding of the underlying demographic structure of study populations, and such data can be obtained using neutral loci from the generated genome-wide data or prior knowledge of a species' phylogeographic history. To this end, we summarize recent simulation studies that test the power and accuracy of genome scan methods under a variety of demographic scenarios and sampling designs. We conclude with a discussion of additional considerations for future method development, and a summary of methods that show promise for landscape genomics studies but are not yet widely used.

## Introduction

Understanding the spatial distribution of adaptive genetic variation is at the very core of evolutionary biology and population genetics. Recent advances in next-generation sequencing make studies of the genomic basis of local adaptation now possible for virtually any organism. Simultaneously, spatial data for nearly every corner of the Earth are available due to dramatic increases in GIS and mapping technologies. These technological developments have led to the rapid proliferation of studies that integrate geographic and genomic data to test for spatial patterns of genes under selection, collectively termed “landscape genomics” (Joost et al., 2007; Lowry, 2010; Manel et al., 2010).

Landscape genomics stems from landscape genetics, an explicitly spatial suite of analysis methods that focus on testing the influence of landscape features on genetic population structure (Manel et al., 2003; Storfer et al., 2007; Manel and Holderegger, 2013). The transition from landscape genetics to landscape genomics has come with the shift from utilizing a dozen or so loci (often microsatellites) to thousands and even millions of loci (often single nucleotide polymorphisms-SNPs)—and even complete transcriptomes or genomes—in studies of spatial genetic variation.

Is landscape genomics just landscape genetics with more loci? In the original article that coined the term “landscape genetics,” Manel et al. (2003) state that, “*Dozens of markers are available for numerous taxa”* and that “*identification of loci under selection can help us understand the genetic basis of local adaptation*…” (p. 190). However, except for candidate gene approaches, where *a priori* information about the function of specific genes is known, dozens of markers are generally insufficient for tests of selection; such tests commonly rely on orders of magnitude more loci to have appropriate statistical power to conduct outlier analyses (Luikart et al., 2003; Pritchard and Di Rienzo, 2010) or genotype-environment associations (i.e., GEAs, Rellstab et al., 2015). As a result, the literature commonly refers to landscape genomics studies as those that (have the power to) focus on describing spatial patterns of selection and adaptation, whereas landscape genetics studies primarily focus on the influence of landscape variables on gene flow (Rellstab et al., 2015; Haasl and Payseur, 2016).

Semantics aside, scientists are now awash with data, and analytical methods have lagged behind our ability to generate massive data sets. The shift from analyzing dozens to thousands to millions of markers (and even whole genomes) brings about new computational challenges. Whereas landscape genetics relies upon a rich history of spatial statistics dating back to the 1950s and 1960s, genome-wide selection analyses have primarily been developed in the last decade. New methods are rapidly being developed, and embarking on a landscape genomics study may seem like a daunting task for some researchers. Here, we aim to disentangle some of the complexity involved in conducting a landscape genomics study and associated downstream analyses, and we hope to offer some perspective for novice and experienced researcher alike. We focus primarily on marker-based studies of non-model organisms, as it is in these systems that landscape genomics studies are most rapidly expanding. Additionally, inference in non-model organisms is particularly challenging as they lack the genomic tools such as reference genomes and transcriptomes, which are typically available for model systems (Manel et al., 2010; Storfer, 2015). We emphasize that this piece is not meant to be an exhaustive review of the subject, as many substantial articles have already been published to this effect (e.g., Haasl and Payseur, 2016; Hoban et al., 2016; Rellstab et al., 2016). Rather, we provide a brief guide to navigate this new and rapidly changing field and in the following sections, we focus on: (1) study design; (2) data generation; (3) analysis methods and associated challenges; (4) methods at the interface of landscape genetics and landscape genomics; and, (5) future directions.

## Study design

Early work in landscape genetics went through an exploratory phase, where sampling was geographically widespread and involved testing the effects of various landscape variables on gene flow and population genetic structure (Storfer et al., 2007, 2010). Similarly, early landscape genomics studies lacked specific hypotheses and were designed to take an unbiased approach to search for candidate loci across populations that differed in key environmental variables (e.g., altitude; Haasl and Payseur, 2016). Instead of using candidate gene or QTL approaches (Stinchcombe and Hoekstra, 2008), tests for selection were conducted across a suite of loci spread throughout the genome without *a priori* information about putative function. High false positive rates are perhaps the most significant problem with landscape genomics studies that rely on genome scans (Lotterhos and Whitlock, 2014, 2015; Rellstab et al., 2015; Haasl and Payseur, 2016), and this is further exacerbated without *a priori* hypotheses. Studies that lack specific hypotheses are prone to choose candidate loci with the strongest associations with environmental variables, with a reasonable chance of detecting spurious result(s). One way to identify false positives is that loci in close proximity do not show a signature of selection. Even if loci detected in such analyses are “true” positives, the function of the candidate loci remains unknown, particularly when lacking a reference genome and thus the ability to map a candidate locus (Pavlidis et al., 2012). Even when a candidate is in linkage disequilibrium with a gene of known function, downstream functional verification may be necessary. Thus, landscape genomics studies should aim to be hypothesis-driven, because inference is stronger when there is documented variation in phenotypes or other specific information that provides evidence of spatial variation in local adaptation among populations.

It is also important to note that landscape genomics studies can test for candidate genes underlying local adaptation, as well as the effects of landscape variables on gene flow. That is, the large number of loci generated for landscape genomics studies can be partitioned into sets of loci that are putatively neutral and those that are putatively under selection, with the former being used to test spatial patterns of gene flow and population structure. Note, however, that sampling designs for assessing population genetic structure and testing for loci under selection have important similarities and differences (Table 1).

**Table 1:** General differences between landscape genetics and landscape genomics studies.

	**Questions**	**Scale of study**	**Sampling design**	**Analysis methods**
Landscape genetics	Influence of landscape on gene flow	Among populations	**Stratified random**, opportunistic, clumped, individual-level	Mantel tests, *Assignment tests* (spatial and aspatial; e.g., Structure, Tess, Geneland), *Ordination (dbRDA, sPCA. MDS)*, Least cost paths (multiple regression, MLPE), Spatial autocorrelation, Spatial regression, EEMS^*^
	Influences of landscape on at-site variation	Within populations	**Across ecological gradients**, stratified	Graph models (e.g, Popgraph), GDMs, Structural equation models
	Barriers	Among populations	**Across hypothesized barrier(s)**	Wombling, Monmonier's maximum difference algorithm, spatial assignment tests (e.g., Geneland)
	Species' ecology	Within and among populations	**Across ecological gradients** (stratified)	Ordination, Least cost paths, Spatial autocorrelation, Spatial regression
	Source-sink dynamics	Among populations	**Across populations of different sizes or fragmentation levels**	Mantel tests, genetic diversity estimates (e.g., F-statistics, bottleneck tests)
Landscape genomics	Spatial patterns of selection	Among populations	**Paired sampling**, transect sampling	Outlier differentiation methods (eg., Bayescan, FLK, X^T^X); Genotype-environment associations (e.g., Bayenv2, PC Adapt, LFMM, sGLMM, Samβada), *Ordination, Assignment tests* (e.g., FASTSTRUCTURE, Admixture, Tess3)
	Influence of landscape on local adaptation	Among populations	Transect sampling, paired sampling, stratified sampling	Outlier differentiation methods; Genotype-environment associations, *Ordination, Assignment tests*, Genomic cline analysis^*^, GDM^*^, EEMS^*^

*Note that, when conducting a landscape genomics study, that when loci under selection are removed and putatively neutral loci remain, that landscape genetics questions and analyses can then be conducted. Nonetheless, sampling designs generally differ between landscape genetics and landscape genomics studies, so some landscape genetics questions may not be addressable in studies with landscape genetics goals. Bolded sampling designs indicate preferred designs for that particular question. Not all analysis methods under each study type are listed, just those that are most commonly used or best suited to address the goals of the study. Note also that assignment test methods generally differ between landscape genetics and landscape genomics studies. Italicized words under analysis type indicate those commonly used in both landscape genetics studies of gene flow and landscape genomics studies of loci involved in adaptation. dbRDA, distance-based redundancy analyses; sPCA, spatial principal components analysis; MDS, multidimensional scaling; MLPE, maximum likelihood of population effects (Clarke et al., 2002); LFMM, latent factor mixed models; sGLMM, spatial generalized linear mixed models; EEMS, Estimated Effective Migration Surface (Petkova et al., 2016). Software names include: Geneland (Guillot et al., 2005), Structure (Pritchard et al., 2000), Tess (Durand et al., 2009), Popgraph (Dyer and Nason, 2004); Bayescan Foll and Gaggiotti, 2008, FLK (Bonhomme et al., 2010), Bayenv2 (Günther and Coop, 2013), PCadapt (Duforet-Frebourg et al., 2014) Faststructure (Raj et al., 2014), Admixture (Alexander et al., 2009), Tess3 (Caye et al., 2016)*.

* *indicates methods not yet widely used but show promise–see Sections Generalized Dissimilarity Modeling (GDM)–Clinal Analyses*.

For both landscape genetics and landscape genomics studies, choosing an appropriate spatial scale for a proposed study area is extremely important. In general, the extent of the study area and spacing of demes within that study area should match the spatial scale of dispersal and thereby the likely scale of environmentally-mediated selection for the study species (Anderson et al., 2010; Richardson et al., 2014; Rellstab et al., 2015; Hoban et al., 2016). Additionally, the resolution of the environmental data should be appropriate for the study species (e.g., sampling at a 2.5 km scale would be inappropriate for a slug species; Anderson et al., 2010). Also, GIS layers chosen for each study should be those deemed to be those most reasonable based on the ecology of the study species and what is known regarding habitat use. However, researchers should be aware that many environmental layers available for analysis in a GIS tend to be multicollinear (e.g., various temperature measures, such as seasonality and maximum temperature). Without some reduction of the number of variables (e.g., via ordination such as PCA), significant relationships between detected between environmental variables and allele frequencies may be spurious and/ or correlated with the true variables. Alternatively, problems with multicollinearity can be avoided by selecting one environmental variable as a representative of each correlated set (e.g., Trumbo et al., 2013). An overview of the use of GIS in landscape genomics studies is provided in Leempoel et al. (2017).

A key difference between landscape studies of gene flow and those designed to detect selection is regarding design of spatial sampling (Table 1). For example, in landscape genetics, when testing hypotheses about effects of a specific environmental variable such as precipitation on population genetic structure, a stratified random design is often preferred (Storfer et al., 2010). In contrast, landscape genomics simulations have repeatedly emphasized that replicated sampling of environmental extremes hypothesized to drive selection (e.g., high and low altitude) results in higher power to detect candidate loci under selection than random sampling or transect designs (De Mita et al., 2013; Lotterhos and Whitlock, 2014, 2015; Rellstab et al., 2015; Stucki et al., 2016; see also Table 2). Nonetheless, transect sampling can be appropriate when populations are expected to be maladapted to extremes, but locally adapted to intermediate conditions (Lotterhos and Whitlock, 2015). Sampling transects can also be useful when sampling across a zone of introgression or when geographic clinal analyses are to be employed (see Section Clinal Analyses). Thus, an important distinction to note between landscape genetics and landscape genomics studies is that the former involves study designs that tend to focus on sampling across environmental variation that should influence gene flow, whereas the latter should most often be designed to sample replicated pairs of populations that experience the same environmental extremes. Replication also helps reduce the chance that candidate loci under selection are false positives; loci detected repeatedly across different environments are less likely to result from confounding effects of population structure or environmental covariances (Rellstab et al., 2015).

**Table 2:** Simulation studies of genome scan methods in landscape genomics.

**Simulation study**	**Study aims**	**Methods tested**	**Demographic models**	**Simulated sampling strategies**	**Selection patterns**	**Major findings**
De Mita et al., 2013	1. Compare methods evaluating differences in type I/II error rates and power 2. Evaluate impact of differences in selection, demography, and sampling strategy on inferences made by genome scans	Logistic Regression (LR; Joost et al., 2007) Generalized Estimated Equation (GEE; Poncet et al., 2010) Coop, Witonsky, Di Rienzo and Pritchard (CWDRP; Coop et al., 2010) Beaumont and Nichols test (FDIST2; Beaumont and Nichols, 1996) Foll and Gaggiotti (FG; Foll and Gaggiotti, 2008) Extended Lewontin and Krakauer (FLK; Bonhomme et al., 2010) Excoffier, Hofer and Foll (EHF; Excoffier et al., 2009) Vitalis, Dawson and Boursot (VDB; Vitalis et al., 2001)	Island Model (IM) Stepping Stone Model (SSM) Hierarchical Model (HM) Selfing + IM/SSM/HM Allogamy	S1-1 individual/population S2–4 individuals/population in 48 regularly sampled populations S3-6 random individuals/population in 12 populations S4-4 random individuals/population in 8 populations as two transects parallel to environmental gradient S5 - 4 random individuals/population in 4 populations sampled at extremes of gradient	None tested	LR and GEE have high FPR (false-postive rates), but fast run time Differentiation-based methods have low FPR, but slow run time Sampling fewer individuals in many populations (10/population for most methods) increases power Under allogamy and IM, all methods are comparable Under allogamy and HM or SSM, differentiation based methods have lower FPR Under selfing and IM, LR sampling using S1 is optimal. Under selfing SSM or HM, LR with S1, BN with S3, and FG with S2 perform best with respective sampling strategies
Frichot et al., 2013	1. Identify signatures of selection controlling for population structure 2. Introduce Latent Factor Mixed Models (LFMM) as a means to test for genetic-environment associations 3. Compare FPR and FDR between methods using spatially explicit neutral coalescent simulations	LFMM (Frichot et al., 2013) LRM (Storey and Tibshirani, 2003) Principle Component Regression (PCRM; Joost et al., 2007) Generalized Linear Models (GLMs; Joost et al., 2007) Standard Linear Mixed Models (GEMMA; Zhou and Stephens, 2012) Partial Mantel Test (PMT; Fumagalli et al., 2011) BayEnv (Coop et al., 2010)	Isolation by Distance (IBD)	None tested	P1 - Correlated with demographic history P2 - Along environmental gradient P3 - Low-intensity selection	LFMM has low FPR under IBD PMTs, LRMs and PCRMs have low power and high FPRs under IBD PMT, PCRM and GEMMA have high FNR when enviornement is strongly correlated with demography LFMM runs faster than BayEnv when analyzing large data sets LFMM performs better then BayEnv when genetic structure well characterized FDR (false-discovery rate) and FPR highly correlated
de Villemereuil et al., 2014	1. Individual-based simulation comparing power and error rates of genome scan methods 2. Characterize role of population structure and mode of selection on outlier detection	Allele frenquency-environmental linear regression (LRM; Storey and Tibshirani, 2003) Bayescan (Foll and Gaggiotti, 2008) BayEnv (Coop et al., 2010) Latent Factor Mixed Model (LFMM; Frichot et al., 2013)	Hierarchical Model (HM) Island Model (IM) Stepping Stone Model (SSM)	None tested	P1 - Correlated with demographic history P2 - Along environmental gradient P3 - Monogenic P4 - Polygenic	Decrease in power in methods under polygenic vs. monogenic selection Under polygenic selection LRM most powerful but has highest FDR BayEnv has low FDR under SSM, high under HM All methods have low power under P1 BayEnv and LRM have highest FPR, LFMM had the most true-positives under P1
Lotterhos and Whitlock, 2014	1. Test effects of IBD and range expansion to detect spatially divergent selection among methods 2. Compare effects of different parameterization and outlier differentiation/GEAs	Beaumont & Nichols test (FDIST2; Beaumont and Nichols, 1996) Bayescan (Foll and Gaggiotti, 2008) Extended Lewontin & Krakauer (FLK; Bonhomme et al., 2010) X^T^X (Günther and Coop, 2013)	Island Model (IM) Isolation by Distance (IBD) Two Refugia (2R) One Refugium (1R)	None tested	Soft selection	Under IBD, FDIST2 and BayeScan have low power and high FPR FDIST2 and BayeScan have low FDR when assumptions of equilibrium are met FLK performs best when no neutral loci or null model is available BayEnv2 has highest power under IBD and non-eq demographic scenarios
Forester et al., 2015	1. Describe how variation in environment, strength of selection and dispersal affect strength of local adaptation 2. Determine which GEAs have the greatest power in competing scenarios	Principial components analysis (PCA) Principial coordinate analysis (PCoA; Bray and Curtis, 1957) Redundancy Analysis (RDA) Distance-based redundancy analysis (dbRDA) Latent Factor Mixed Model (LFMM; Frichot et al., 2013)	IBD with varying dispersal distances: 5% 10% 15% 25% 50%	None tested	P1 - Continuous (clinal) gradient P2 - Discrete spatial selection with habitat aggregation (10%) P3 - Discrete spatial selection with habitat aggregation (50%) P4 - Discrete spatial selection with habitat aggregation (90%)	RDA and dbRDA have highest power, low FPRs and strongest GEA indices under all scenarios PCA, PCoA & LFMM show stronger GEA indices at intermediate dispersal levels Ordination methods broadly control for population structure due to IBD better then other techniques Changes in habitat aggregation and selection have small effects on spatial structure at neutral sites
Lotterhos and Whitlock, 2015	1. Compare power of GEAs and outlier differentiation methods to detect loci involved in local adaptation based on: Sampling design and 2. Demography	X^T^X (Günther and Coop, 2013) PCAdapt (Duforet-Frebourg et al., 2014) BayEnv2 (Günther and Coop, 2013) Latent Factor Mixed Model (LFMM; Frichot et al., 2013)	Island Model (IM) Isolation by D istance (IBD) Two Refugia (2R) One Refugium (1R)	S1 - Transect S2 - Paired sampling S3 - Random	Weak clinal selection	Pairwise sampling have high power for detecting genes under weak selection, transects better at detecting clines Total sample size influenced power more than distribution of populations LFMM has higher power then Bayenv2 with more samples, but higher FPR LFMM and Bayenv2 have high power because they explictly account for relatedness and environment

*Summarized are questions, sampling methods, analysis methods and conclusions as to which methods lead to low false positive rates and high power to detect loci under selection*.

With limited resources, researchers generally face a tradeoff between the total number of samples and the total number of localities that can be sampled in genetics studies of natural populations. Landscape genetics study designs often focus on maximizing the number of individuals per location to obtain accurate allele frequency estimates (Storfer et al., 2010; Manel and Holderegger, 2013). Most landscape genetics analyses are genetic distance-based, and inaccurate estimates of allele frequencies can bias gene flow estimates (Storfer et al., 2007, 2010). While replication of sites or transects is favored for reasons above in landscape genomics studies, the balance between sample size and number of sites depends on downstream analysis type. Power is generally limited by the total number of samples collected in landscape genomics studies (Lotterhos and Whitlock, 2015). Indeed, it is important to sample a sufficient number (e.g., > 10) of individuals per locality to generate accurate allele frequency estimates for analyses that rely on estimates of genetic differentiation among populations (i.e., differentiation outlier analyses below). However, optimizing the number of population pairs sampled (with smaller sample sizes per location) can be robust for detecting selection when sampling locations represent a range of environmental variable values across the study area (De Mita et al., 2013; Table 2).

## Data generation

Initially, landscape genomics studies expanded from microsatellites commonly employed in landscape genetics studies to a few hundred AFLPs (amplified fragment-length polymorphisms; Joost et al., 2007). Currently, landscape genomics studies typically rely on genome-wide SNP marker sets generated using short-read next generation sequencing technologies (e.g., Illumina). Perhaps the most widely used of such reduced-representation approaches in the last few years is RAD-seq (restriction-associated digest DNA sequencing; Andrews et al., 2016; Lowry et al., 2017). RAD-seq is particularly appealing because it does not rely on availability of a reference genome. In short, whole genomic DNA is cut into fragments using a restriction enzyme, sequencing bar codes are ligated to restriction sites, individuals are bar-coded and fragments are sequenced using next-generation technology (Andrews et al., 2016). Homologous fragments among individuals are aligned (e.g., using Stacks Catchen et al., 2013 or other software), and thousands to millions of SNPs are identified. RAD-seq has been extremely beneficial for studies of population genetic structure, as well as pedigree and other analyses (Andrews et al., 2016; Catchen et al., 2017). Therefore, RAD-seq can be a powerful approach for landscape genetics studies. As with other genotyping-by-sequencing methods, RAD-seq, while beneficial for genotyping large numbers of individuals, suffers from marker attrition. That is, the more individuals sequenced, the fewer loci become available for robust analyses due to genotyping errors due low coverage or missing data. Additionally, a shortcoming of RAD-seq for landscape genomics studies is that generally only a small fraction of a genome is sampled, and thus loci involved in adaptation are often missed (Lowry et al., 2017). Further, without a reference genome, identified SNPs are anonymous, and downstream work is necessary to determine their function (Lowry et al., 2017).

As a potential solution, transcriptome sequencing and exome capture are reduced representation approaches that focus on genic (i.e., coding) regions. Genes will contain much of the functional genetic variation that underlies adaptation, and such regions are also in linkage with promoter regions also under selection (Hoekstra and Coyne, 2007; Stern and Orgogozo, 2008). RNA-seq is an approach to sequence total RNA or the mRNA transcriptome, which can be used to evaluate gene expression levels (in different environments) and, when multiple transcriptomes are sequenced, SNPs can be identified. A series of capture probes can then be designed to sequence the flanking region around identified SNPs in cDNA. Assembled transcriptomes, can then be used to annotate functional information for candidate SNPs since they are all found in coding DNA. Further, when SNP codon positions are identified, traditional sequence-based population genetic tests for selection can be applied (e.g., MK test; McDonald and Kreitman, 1991 or *dN*/*dS* ratios). Transcriptome sequencing, however, will only capture a subset of all coding genes, as gene expression is tissue-specific (Bishop et al., 1974). Exome capture sequencing will increase the number of coding loci (Jones and Good, 2016).

Another method used for genome-wide marker generation in non-model species is Pool-seq (reviewed Schlötterer et al., 2014), whereby a large number of individuals (dozens to hundreds) are pooled and sequenced together. Advantages include reduced cost, and genome-wide data generation that facilitates SNP identification and allele frequency generation for population genetic analyses. Disadvantages include lack of ability to identify individual samples, difficulties identifying rare variants, and potential alignment issues owing to non-homologous sequences (i.e., paralogs), and lower confidence in SNP assignment than other methods (Schlötterer et al., 2014). Software such as PoPoolation (Kofler et al., 2011) can help account for some of the bias introduced by pooling and sequencing errors. Nonetheless, pool-seq works much better when a reference genome is available and short-read sequences can be aligned and mapped to reduce alignment errors among pools. Even with a reference genome, structural variation (e.g., inversions, indels) between pooled resequenced samples and the reference can generate falsely identified SNPs (Tiffin and Ross-Ibarra, 2014).

## Analysis considerations

Similar to landscape genetic studies, there is a wide array of analysis methods for landscape genomics analyses and new methods are continuously being developed (Hoban et al., 2016). The key difference between the two analytical frameworks is that landscape genetics studies rely on use of putatively neutral markers to generate estimates of genetic population structure, whereas tests of selection in landscape genomic studies generally require the need to control for population structure (see Table 1). As above, note that genome-wide marker sets generated for landscape genomics tests of selection can also be parsed into neutral data and landscape genetics analyses can be employed (see Storfer et al., 2007, 2010; Guillot et al., 2009; Shirk et al., 2017). Landscape genomics studies employ tests for loci under selection using genome scans, candidate gene approaches, quantitative trait locus mapping and genome-wide association studies (see Stinchcombe and Hoekstra, 2008; Storfer, 2015). However, genome scans are the most widely used, as the latter analysis types tend to be used for model systems. It is important to note that numerous excellent reviews (e.g., Rellstab et al., 2015; Haasl and Payseur, 2016; Hoban et al., 2016) discuss in detail the benefits and limitations of the various genome scan methodologies and associated software. As such, we summarize the main considerations here.

Genome scans generally use two approaches to detect loci under selection: (1) differentiation outlier methods (which were previously called F_ST_-outlier tests, but now include other methods of genetic differentiation among populations; Hoban et al., 2016); and, (2) genetic-environment association (GEA) tests (Schoville et al., 2012; Pardo-Diaz et al., 2015; Rellstab et al., 2015; Hoban et al., 2016). Differentiation outlier methods rely on the demonstration that, at migration-drift equilibrium under a neutral island model with spatially uniform migration and gene flow, population differentiation of allele frequencies (e.g., F_ST_) across a large number of loci can be used to infer the process of selection acting on a subset of loci (Lewontin and Krakauer, 1973). Statistical outlier loci with significantly greater F_ST_ (or other genetic distance) values than the distribution of genome-wide F_ST_ values are presumed to be under diversifying or local selection or linked to those under selection (Black et al., 2001; Luikart et al., 2003). Similarly, loci with significantly lower F_ST_ values are inferred to be under stabilizing or purifying selection (Black et al., 2001; Luikart et al., 2003). Thus, unlike landscape genetics studies which generate genetic distance estimates among a small number of loci to elucidate effects of landscape variables on gene flow, landscape genomics studies rely on a very large number of loci to generate a frequency distribution of genetic distance values as a null against which to test for outliers under selection.

Early methods to conduct such outlier tests include FDIST (Beaumont and Nichols, 1996; implemented in LOSISTAN) to identify strong differences from the null distribution of F_ST_ values across loci. Later, the widely used BayeScan (Foll and Gaggiotti, 2008) was developed, which uses a Bayesian method to estimate the relative probability that each locus is under selection. PCAdapt is a recently developed popular method that uses a principal components analysis framework to detect candidate loci under local adaptation (Duforet-Frebourg et al., 2014). Methods that use genetic distance measures other than F_ST_ include FLK (Bonhomme et al., 2010), which uses a modified version of the Lewontin and Krakauer (1973) test for selection by comparing allele frequencies of different populations in a neighbor-joining tree constructed using a matrix of Reynold's genetic distance (Reynolds et al., 1983), and X^T^X, which employs a Bayesian method to test individual SNPs against a null model generated by the covariance in allele frequencies between populations from the entire set of SNPs (utilized in Bayenv2; Coop et al., 2010; Günther and Coop, 2013). Summaries of differentiation outlier methods can be found in Hoban et al. (2016; Appendix 1). Notably, differentiation outlier methods are aspatial in nature.

GEAs (also referred to as EAAs or environmental association analyses; Rellstab et al., 2015) are spatial because they are designed to test for significant correlations between allele frequencies at particular loci with variation in environmental variable(s) (Joost et al., 2007; Hancock et al., 2011; Rellstab et al., 2015). Thus, unlike differentiation outlier approaches, GEAs require availability of environmental data from sources such as WorldClim data (http://www.worldclim.org, Hijmans et al., 2005). Widely used methods include Bayenv2, which tests for GEAs in addition to differentiation outliers, and latent factor mixed models (LFMM; Frichot et al., 2013). Bayenv2, tests for large allele frequency differences across environmental gradients by comparing observed allele frequency differences to transformed normal distribution of underlying population frequencies. Latent factor mixed models (LFMM; Frichot et al., 2013), include population structure as latent (or hidden) variables to limit false positive signals. Spatial generalized linear mixed models (SGLMMs; Guillot et al., 2014) are an extension to LFMMs and have proven to be computationally more efficient. Ordination approaches, such as redundancy analysis, can also be used in GEAs (Forester et al., 2015); ordination is also widely used in landscape genetics studies (Storfer et al., 2010). Another more recently developed GEA method is Samβada (Stucki et al., 2016), which is a multivariate analysis framework that accounts for underlying population structure with estimates of spatial autocorrelation in the data. To search for loci under selection, Samβada uses linear regressions to model the probability of observing a particular allele given the value of environmental variables at the location it was sampled for each locus independently (Stucki et al., 2016). A summary of GEAs and their assumptions can be found in Rellstab et al. (2015; Table 1).

### Analysis concerns

Fundamentally genome scan methods operate on the assumption that loci under selection can be differentiated from a null distribution of allele frequencies generated by neutral processes. Determining how much genetic differentiation can be expected in populations in the absence of selection, however, remains a great challenge (Lotterhos and Whitlock, 2014; Hoban et al., 2016). Thus, the primary concern with employing genome scan analyses is differentiating false positive signals from loci that are actually under selection.

Underlying population demographic structure, when not properly accounted for, can be a principal source of false positives. There are several demographic scenarios that can generate neutral allele frequency differentiation among populations that can falsely be interpreted as signals of selection (Lotterhos and Whitlock, 2015; Rellstab et al., 2015; Haasl and Payseur, 2016). A straightforward example is illustrated by the case of allele surfing, whereby serial population bottlenecks that occur during founder effects of small populations migrating to new areas can result in fixed allelic differences among populations that are solely due to genetic drift (Excoffier et al., 2009; Waters et al., 2013). Similarly, recent population range expansions from refugia can generate correlations between allele frequencies and environmental variables that are not due to selection. In general, landscape genomics studies are challenging in small, patchy populations that are prone to genetic drift, which can result in the appearance of spatially distributed loci under selection. False signals of selection can also be generated by locus-specific hybridization or introgression from related taxa (Fraïsse et al., 2016; Hoban et al., 2016). Nonetheless, in cases where selection gradients follow the same spatial pattern as background genetic population structure, candidate loci under selection can be missed due to false negative signals.

In general, demographic structure can influence the null distribution of F_ST_ or other genetic differentiation measures and thereby bias significance testing (Lowry, 2010; Whitlock and Lotterhos, 2015). Each genome scan method utilizes a different way to account for underlying population demography. For example, FDIST assumes populations follow an island model (Beaumont and Nichols, 1996) to generate null F_ST_ distribution. The recently developed OutFLANK (Whitlock and Lotterhos, 2015), however, does not invoke a specific demographic model. Rather, OutFLANK infers the distribution of F_ST_ for loci unlikely to be strongly affected by spatially diversifying selection (Whitlock and Lotterhos, 2015). Specifically, OutFLANK uses a modified Lewinton-Krakauer method to infer a null F_ST_ distribution, which approximates a χ^2^ distribution with adjusted degrees of freedom. Then, differentiation outliers are identified as those that fall outside this trimmed, putatively null F_ST_ distribution.

Approaches that use covariance matrices or linear models to account for population structure are also flexible because they have no explicit underlying population demographic model. For example, Bayenv2 is a GEA method that controls for genetic population structure in by generating a variance-covariance matrix of relatedness among samples; candidate loci are determined as those for which an environmental variable explains significantly more variation than the variance-covariance matrix of all other loci (Günther and Coop, 2013). Linear model approaches, such as LFMMs and SGLMMs, can limit false positives in both GEAs and outlier tests by including population structure as latent variables (Frichot et al., 2013; Lotterhos and Whitlock, 2015). Samβada uses estimates of underlying spatial autocorrelation in genetic data as a way to control for underlying population structure (Stucki et al., 2016).

A number of informative simulation studies that explore the power of the different methods under different demographic or other scenarios have recently been published (De Mita et al., 2013; Frichot et al., 2013; Jones et al., 2013; de Villemereuil et al., 2014; Lotterhos and Whitlock, 2014, 2015; Forester et al., 2015; See Table 2 for a summary of the study conditions and their findings). The relative power of GEAs and differentiation outlier tests is dependent on the underlying demographic model. GEAs have higher power under an island model, whereas outlier tests have higher power under an isolation-by-distance model (Lotterhos and Whitlock, 2015). Within GEAs, the degree of patchiness in the landscape affects the power and false positive rates (Forester et al., 2015). With limited dispersal and strong isolation-by-distance, univariate GEAs had high false positive rates (FPRs; up to 55%) and constrained ordination procedures (e.g., redundancy analyses, or RDA) performed much better with lower FPRs (0–2%; Forester et al., 2015). Within outlier differentiation methods, Bayenv2 and FLK outperformed FDIST and Bayescan for systems experiencing IBD and recent range expansions (Lotterhos and Whitlock, 2014). Of all GEAs and outlier detection methods, LFMMs were generally found to have relatively low false positive rates (Type I error rates) than other methods (Jones et al., 2013; Joost et al., 2013).

Even after accounting for the underlying population structure, however, there are other important considerations that can affect the power of genome scan studies and their interpretation. To date, no methods have been developed to account explicitly for background selection (Hoban et al., 2016), which can result in population diversification due to purifying and not positive selection (Charlesworth et al., 1993). Background selection can thus cause errors in estimating the null distribution and thereby reduce power of genome scans (Tiffin and Ross-Ibarra, 2014; Haasl and Payseur, 2016). Signatures of local adaptation can also be incorrectly inferred as a result of spatially uniform positive selection. That is, across landscapes with limited gene flow, multiple beneficial mutations may arise to reach an optimal phenotype, resulting in a patchwork of allele frequencies. This can result in detectable genetic differentiation across the patches that produces false signals of selection by local environment (Hoban et al., 2016).

It is also important to note that genome scan analyses are biased to detect large effect loci, because power to detect small effect loci is generally low (Pritchard and Di Rienzo, 2010). Because most phenotypic traits are likely to be polygenic, and thus governed by many loci of small effect (Rockman, 2012), genome scan methods are prone to miss most loci involved in local adaptation (Stephan, 2015). Further, the polygenic nature of phenotypic traits means candidate loci explain a small proportion of phenotypic variation, which has been termed the “missing heritability problem” (Hindorff et al., 2009; Visscher et al., 2010; Yang et al., 2010, 2012). Recently, multilocus approaches have been developed that quantify the strength of selection acting on correlated loci using Bayesian sparse linear mixed models (Gompert et al., 2017). However, these approaches necessitate large sample sizes and time-series sampling, thereby limiting their widespread applicability. In addition, for studies that employ anonymous SNP markers when no reference genome exists, such as RAD-seq, candidate genes are assumed to be in linkage disequilibrium (LD) with loci under selection and are most often not under selection themselves (Lowry et al., 2017). With a reference genome, estimates of LD decay can be used to determine the size of the window to search for possible genes linked to a candidate SNP detected in a genome scan when the SNP is not in a gene itself. However, we do not know the extent of LD for most species, and the size of LD blocks is not constant throughout the genome (Tiffin and Ross-Ibarra, 2014; Lowry et al., 2017). These factors can make mapping and annotating candidate markers prone to error.

### Combinatorics and other multivariate approaches

An important consideration in landscape genomics studies is how to integrate data analyses across multiple genome scan methods. One fairly standard approach is to construct Venn diagrams and use combinatorics as a method of validation for candidate loci. That is, the larger the number of genome scan methods that detect a particular candidate locus under selection, the more confident researchers tend to be that the candidate is truly under selection. However, genome scan methods each have different assumptions and different power to detect loci under selection, depending on population demography, sampling design and nature of the selective sweep (Lotterhos et al., 2017). Thus, reliance on concordance of multiple univariate methods to prioritize loci for further research is prone to miss loci under weak selection (Lotterhos and Whitlock, 2015).

Recent proposed solutions have included multivariate methods that combine *P*-values and control for false discovery rates (FDR; Benjamini and Hochberg, 1995). For example, de-correlated composite of multiple signals (DCMS) controls for genome-wide correlations among statistics by weighting each locus depending how correlated a particular statistic that detected the locus is to other statistics (Ma et al., 2015). Thus, the less a test statistic is correlated to another statistic(s), the higher the locus is weighted. François et al. (2016) built on earlier methods to control for FDR (e.g., Benjamini and Hochberg, 1995) using a “genomic inflation factor” to adjust the distribution of *p*-values. In general, composite methods tend to perform better than univariate methods, but their performance has only been evaluated in a narrow set of circumstances (Lotterhos et al., 2017).

Even newer methods include analyses to filter, visualize and integrate multiple univariate analyses in multivariate space (Lotterhos et al., 2017; Verity et al., 2017). For example, MINOTAUR (Multivariate vIsualizatioN and OuTlier Analysis Using R) is a program that uses one of four different distance measures (Mahalanobis distance, harmonic mean distance, nearest neighbor distance and kernel density deviance) to test the significance of loci (Verity et al., 2017). An important future direction is to continue to evaluate the variety of methods for evaluating and prioritizing candidate loci for future research. As we learn more about the genomic architecture of different species, we can continue to test the performance of existing methods, or develop new methods as appropriate.

### Analysis considerations-summary

In general, researchers should avoid the temptation to analyze their data with as many genome scan methods as possible. Instead, several factors that should be considered when choosing genome scan method(s) to be employed. First, if attainable, knowledge of underlying demographic structure can be used to choose the most powerful methods that are least prone to Type I errors for that specific demographic history. For example, phylogeographic analyses can be used to assess whether there have been recent geographic range expansions from glacial refugia. To parameterize the number of latent factors (e.g., in LFMM or SGLMM), the number of genetic clusters (*K*) could be determined using a Bayesian clustering algorithm such as FastSTRUCTURE (Raj et al., 2014) or ADMIXTURE (Alexander et al., 2009). Note that incorrect assumptions about underlying demographic structure can increase both Type I and Type II error (Pérez-Figueroa et al., 2010; Jones et al., 2013; Lotterhos and Whitlock, 2014), and in such cases, model-free approaches may be preferred. Second, given the numerous additional concerns for which researchers have little ability to estimate (e.g., variation in genome-wide LD) or control for (e.g., the polygenic nature of most phenotypic traits), confidence in candidate loci as real targets of selection comes from their repeated detection across replicated transects or paired sampling locations. Similarly, candidate loci detected by multiple analysis methods also decreases the likelihood that they are false positives. Third, as stated above, inference of candidate loci is improved when selective agent(s) are known before embarking on a landscape genomics study. Candidate genes identified in genic pathways that influence particular phenotypes known to be under selection are less likely to be false positives than randomly detected loci or those without known function.

## Methods at the interface of landscape genetics and landscape genomics

### Generalized dissimilarity modeling (GDM)

Originally used to model species community turnover (Ferrier et al., 2007), GDMs have recently been adopted for use in landscape genetics studies. GDMs involve fitting I-splines that are monotonic, nonlinear functions that, when rescaled between 0 and 1, represent importance of environmental variables in explaining turnover of allele frequencies (Fitzpatrick and Keller, 2015). GDMs have been used to assess effects of at site environmental differences on gene flow (also called “isolation by environment”; Wang and Bradburd, 2014). I-splines can be non-linear, providing an advantage over linear approaches because they may be able to identify threshold values (i.e., the point along the environmental axis where the slope of the spline is greatest) for landscape variables. Similarly, GDMs can be applied to landscape genomics studies by fitting I-splines to the relationships of ecological variables on allele frequencies at putatively adaptive loci. Related to GDMs, which employ distance-based measures are gradient forests, an extension of random forests, which both employ machine-learning algorithms for model optimization (Breiman, 2001). Similar to GDM, gradient forests fit nonlinear monotonic functions to characterize allele-frequency turnover across environmental gradients for each locus independently (see Fitzpatrick and Keller, 2015). As such, both approaches can be used to identify a loci with high degree of allelic turnover associated with specific environmental variables, and thus yield candidate loci under selection.

### Estimated effective migration rate

Another recently developed method that can be applied to both landscape genetics and landscape genomics studies is the Estimated Effective Migration Surface (EEMS: Petkova et al., 2016). This method differs from other approaches that identify underlying population demographic structure (e.g., clustering and PCA-based approaches), because genetic differentiation is modeled as a function of estimated migration rates. EEMS uses a stepping stone model (Kimura and Weiss, 1964) that allows for migrations of variable rates to occur among a set of demes. This process is modeled by overlaying a dense regular grid over the study area and calculating an approximation of the expected genetic dissimilarity through the use of resistance distance, similar to “isolation-by-resistance” (McRae, 2006). Consequently, areas in which genetic dissimilarity decays more slowly will be assigned a greater value of Effective Migration Rate (EMR), than those for which genetic dissimilarity decays more rapidly.

EEMS offers two potential applications to landscape genomics studies. First, it can allow researchers to detect underlying demographic population structure, which can be used to help reduce false positive rates in genome scan methods. Second, EEMS analyses could be run separately on data sets containing only putatively neutral or putatively adaptive loci, and can then be used to visualize geographic features that impede gene-flow of neutral or adaptive loci, respectively.

### Clinal analyses

Clines have a rich history in population genetics and bridge both at-site and between-site analyses used in landscape genetics and genomics. To date, most clinal analyses on genome-scale data have focused on the study of hybrid zones and the detection of differential introgression (Gompert and Buerkle, 2010, 2011, 2012). While originally developed for use in identifying loci involved in adaptive divergence and reproductive isolation among hybridizing lineages, genomic cline models could be applied to identify candidate loci for population pairs for which a genome-wide admixture gradient (e.g., via ADMIXTURE or another assignment-based program) has been identified. Loci for which genomic clines possess outliers in one or both of these cline parameters may be subject to selective forces. Outlier loci with alleles introgressing most slowly can be interpreted as those involved in differential adaptation among populations, whereas loci introgressing most rapidly are likely to be uniformly advantageous.

**Figure 1:**
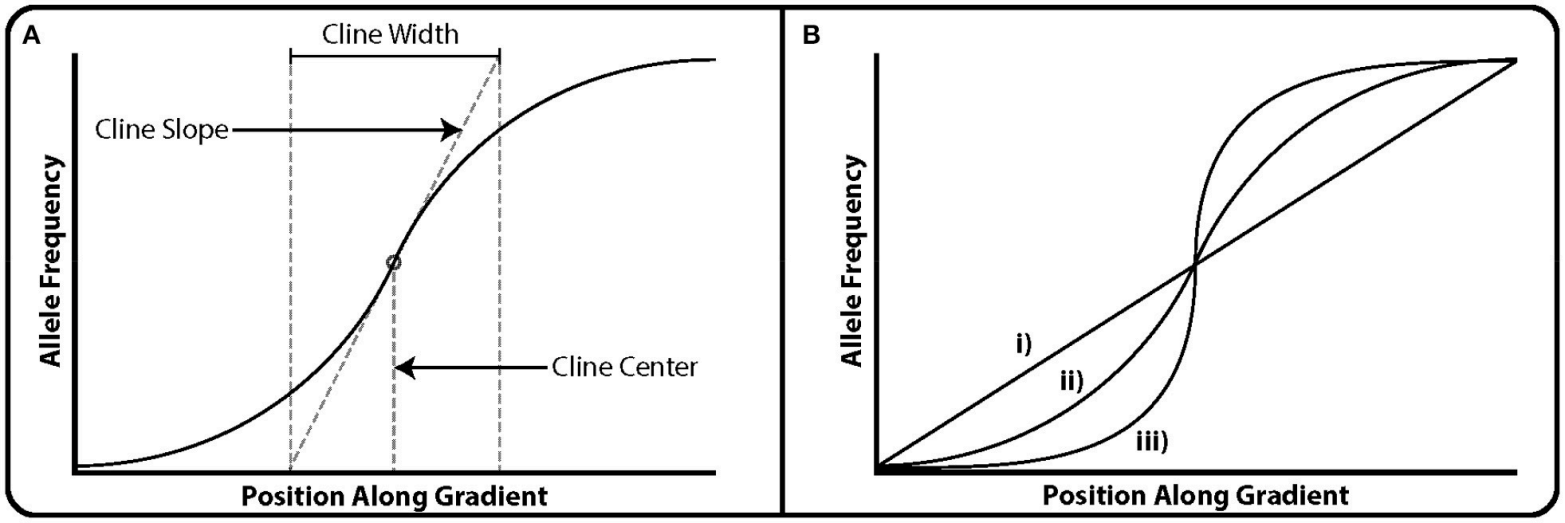
An illustration of clines. X-axes correspond to position along geographic transects (ecological gradient) or hybrid indexes (genomic gradient) in the case of genomic cline analyses. **(A)** Illustration of the three parameters typically estimated in the use of geographic of genomic cline analysis. Cline slope is the estimate of the rate of allele frequency turnover at the steepest point in the cline. In genomic cline analysis this corresponds to the rate of introgression. Cline center corresponds to the point along the geographic transect or hybrid index at which allele frequency turnover is greatest. Cline width corresponds to the region along the gradient at which it's influence on allele frequency is greatest. **(B)** Three examples of clines. (i) A transect along which no selection appears to be acting, or the effects of gene flow are such that changes in allele frequency are purely a function of distance. In the case of genomic cline analyses, the loci under consideration appears to be favored equally in both parental taxa. (ii) A modest cline in which the allele favored by selection changes along the gradient. Given its shallower slope, selection may either be weaker, gene flow stronger (in the case of geographic transects) or the ecotone separating ends of the transect greater. (iii) A steep cline, often called a step cline. In the case of geographic clines, these are formed either by strong selection acting in favor of one allele along a sudden ecotone, or extremely limited gene flow along said ecotone. In the case of genomic clines, this may be due to heterozygote disadvantage, as in the case of reinforcement.

Geographic cline models can explicitly measure the strength of selection on a locus, given the shape of a cline (Endler, 1977; Slatkin, 1987). Geographic cline analyses involve fitting a sigmoidal *tanh* cline model to allele frequencies and quantitative data such as environmental data or a measure of geographic distance (Figure 1; Szymura and Barton, 1986, 1991). Then, cline center, width and slope are estimated along a geographic transect (requiring transect sampling). GEAs are essentially clinal analyses but focus only on the slope of the cline between sampling locations. However, geographic cline analyses analyze the shape of the cline; selection tends to steepen the cline, gene flow widens and reduces the steepness of the cline, and genetic drift narrows the cline (Figure 1; Endler, 1977; Nagylaki, 1978). Researchers can then compare the shapes of observed allele frequency clines in putatively adaptive loci to the shape of clines for neutral loci, as well as those predicted by models of pure migration or drift (Nagylaki, 1978). Unfortunately, current implementations of geographic cline models (e.g., *Analyse*: Barton and Baird, 1995; *hzar*: Derryberry et al., 2014) are computationally burdensome, thus limiting cline fitting to datasets with small numbers of loci. Therefore, geographic cline analysis is currently best suited for use with a reduced set of candidate loci as identified by genome scans.

## Future directions

In the future, landscape genomics should integrate analyses on two scales—the landscape of the genome, and the ecological landscape. Specifically, the landscape of the genome refers to overall genomic architecture, such as the arrangement of loci on chromosomes, placement of inversions, deletions and copy number variants. All of these, ultimately, can affect gene expression, which is further modified by the environmental context in which an individual exists. However, the current state of landscape genomics studies is primarily to generate a list of candidate loci under selection, and, when possible annotate genes in LD with identified SNPs or other genetic variants. Nonetheless, scientists are increasingly aware that the genotype-phenotype relationship is influenced by far more of the genome than just genic sequences. For example, copy number variation and not sequence variation that determines how much human amylase, responsible for starch digestion, is expressed in saliva (Perry et al., 2007). Selection has acted on copy number variation in the amylase gene (*AMY1*) in the human populations; those with high starch diets have higher numbers of copies than populations with diets lower in starch (Perry et al., 2007). Similarly, camels have the highest number of copies known (11) of the *CYP2J* gene (related to salt homeostasis) likely due to selection for high salt tolerance necessary in desert environments (Wang et al., 2012). Transposable elements, which comprise over half the genome of many eukaryotes, were once thought of as parasitic or “junk” DNA (Federoff, 2012). However, evidence suggests that transposable elements are maintained in eukaryotic genomes due to their heritable role in epigenetic mechanisms, such as gene silencing (Federoff, 2012). DNA methylation patterns also influence gene expression and can also be heritable (Anway et al., 2005; Skinner et al., 2012). Promoters and other regulatory regions are also key determinants of gene expression levels and consequently phenotypes. Further, genes are expressed differently in different ecological environments, and selection varies spatially across the ecological landscape. In summary, genomic architecture plays a significant role in the genotype-phenotype relationship, as evidenced by the fact that “large effect SNPs” tend to explain a small fraction of phenotypic variation in natural populations (Hindorff et al., 2009; Rockman, 2012).

Given that technological advances continue to make whole genome sequencing more and more feasible in terms of cost and computational speed for genome assembly, a key challenge for the future of landscape genomics will be the development of methods that integrate multiple data types. Difficulties will include: (1) accounting for the effects of coding and non-coding regions of genomes and overall genomic architecture, combined with protein expression levels, on phenotypic variation; (2) coding for genomic features such as copy number, chromosome inversions or transposable element composition or location in our population genetic models (i.e., Can they be considered in the same way as alleles?); (3) constructing hierarchical models to integrate sources of error from different data types. Then, the challenge is compounded further with the necessity to integrate these complex genomic models with multiple types of spatial environmental data and habitat models in ways that optimize sampling while avoiding potential biases. Mapping the genotype-phenotype relationship has been a key challenge for evolutionary biology for over a century, and landscape genomics will provide the analytical framework to do so across spatially variable ecological environments. A long road may lie ahead, but it is certainly an exciting time for landscape genomics to unravel the complexity of the genomic architecture that underlies local adaptation.

## Conclusions

Landscape genomics has emerged as a prominent framework for studying the genomic basis of local adaptation. Using large genomic data sets, researchers scan the genome for loci that exhibit signatures of selection across heterogeneous environments (Haasl and Payseur, 2016). These efforts have been highly successful, for example, in identifying genes underlying hypoxia adaptation in high-elevation human populations (Beall, 2007a,b; Simonson et al., 2010), environmental responses in Oak populations along climatic gradients (Sork et al., 2016), and differences in growth response amongst Salmon populations in response to geological conditions (Vincent et al., 2013). Studies of biotic factors, have also successfully in identified local adaptation to life history traits (Sun et al., 2015), community composition (Harrison et al., 2017), and disease prevalence (Leo et al., 2016; Mackinnon et al., 2016; Wenzel et al., 2016). Landscape genomics has already dramatically helped to further our understanding of the genomic basis of adaptation (Funk et al., 2012; Shryock et al., 2015). Here, we suggest the field can advance with a careful consideration of explicit hypotheses that, in turn, guide study design, and employment analysis methods that help control confounding factors such as underlying demographic structure. Future landscape genomic research will better integrate genomic architecture in assessments of candidate loci under selection.

## Author contributions

AS conceived of, and wrote most of the paper. AP and AF contributed to the writing, as well as gathered information for, and assembled Table 2.

### Conflict of interest statement

The authors declare that the research was conducted in the absence of any commercial or financial relationships that could be construed as a potential conflict of interest.
